# Multimodal Imaging in Pachychoroid Neovasculopathy: A Case Report

**DOI:** 10.4274/tjo.89166

**Published:** 2018-10-31

**Authors:** Özlem Biçer, Figen Batıoğlu, Sibel Demirel, Emin Özmert

**Affiliations:** 1 Ankara University Faculty of Medicine, Department of Ophthalmology, Ankara, Turkey

**Keywords:** Pachychoroid neovasculopathy, choroidal neovascularization, optical coherence tomography angiography

## Abstract

Pachychoroid neovasculopathy (PNV) is a form of type 1 neovascularization characterized by dilated choroidal vessels in areas of increased choroidal thickness. In this article, we describe a patient diagnosed with PNV. A 50-year-old male with a 2-month history of blurred vision was referred to our clinic. His best corrected visual acuity was 20/100 in both eyes. Retinal pigment epithelium alterations, which were more prominent in fundus autofluorescence, were detected in both eyes on dilated fundus examination. Characteristic findings of PNV were detected in fundus fluorescein angiography, indocyanine green angiography, spectral domain optical coherence tomography, and optical coherence tomography angiography.

## Introduction

Pachychoroid spectrum diseases were first recognized in 2013 when Warrow et al.^1^ described pachychoroid pigment epitheliopathy. The pachychoroid spectrum includes 4 disease groups: pachychoroid pigment epitheliopathy, central serous chorioretinopathy, pachychoroid neovasculopathy (PNV), and polypoidal choroidal vasculopathy. Pachychoroid spectrum diseases are characterized by increased choroidal thickness, dilation of the outer choroidal veins (pachy-veins), and thinning of Sattler’s and choriocapillaris layers.^2^

Multimodal imaging methods are used to understand the disease pathophysiology and in diagnosis. Indocyanine green angiography (ICGA) is shown to be superior to fundus fluorescein angiography (FFA) for detailed imaging of choroid neovascularization (CNV) and diagnosis of choroidal polyps. Thanks to its longer wavelength, ICGA enables better visualization of lesions underlying the retinal pigment epithelium (RPE), even in the presence of blood, exudate, and pigment epithelium detachment (PED).^3,4^ Advances in the field of optical coherence tomography (OCT) have also enabled imaging of choroidal structures in addition to the retina.^5,6^ OCT angiography (OCT-A), a relatively new technology, provides structural information about the retinal and choroidal vessels without the need for contrast material injection.^7^

In this case report, we analyze the findings obtained with various imaging modalities from a patient with PNV who presented with a 2-month history of blurred vision.

## Case Report

A 58-year-old male patient with no other known disease presented to our clinic with blurred vision for the last 2 months. His best corrected visual acuity was 20/100 in both eyes. Pupils were isochoric and light reflexes were present bilaterally. There was no afferent pupillary defect. Slit-lamp anterior segment examination was normal and intraocular pressure values were within normal limits. Fundus examination revealed RPE changes in the macula of both eyes.

**Figure 1:**
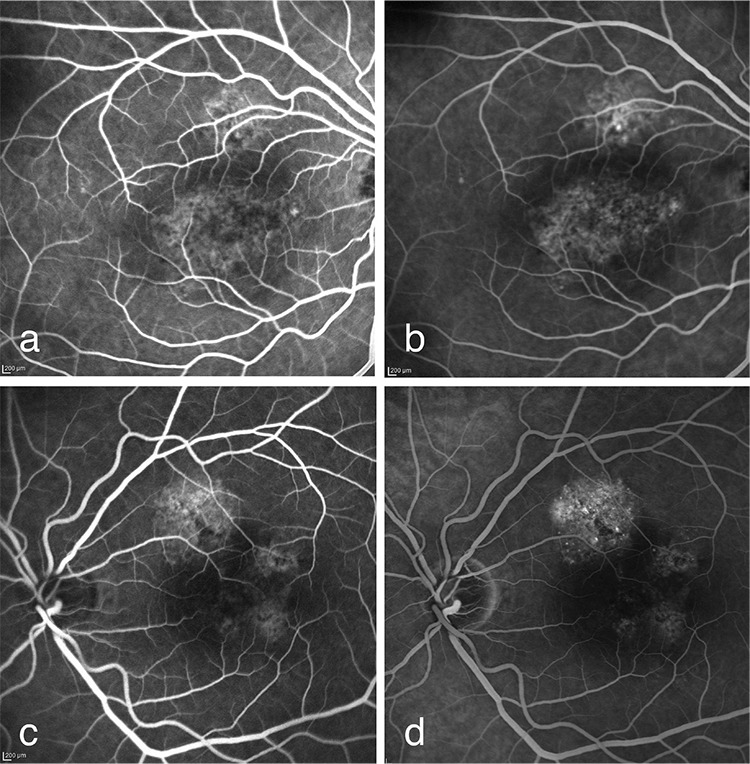
a) Faint hyperfluorescence in the central and superior parafoveal area starting at second 28 of fundus fluorescein angiography in the right eye; b) irregular hyperfluorescence in the central and superior parafoveal area continuing at 4 minutes, 32 seconds of fundus fluorescein angiography in the right eye; c) irregular hyperflorescence in the parafoveal areas at second 37 of fundus fluorescein angiography in the left eye; d) irregular hyperflorescence in the parafoveal areas continuing at minute 5 of fundus fluorescein angiography in the left eye

Irregular hyperfluorescent areas were observed in both eyes in the early and late phases of FFA (Heidelberg retinal angiograph 2) (Figure 1).

**Figure 2:**
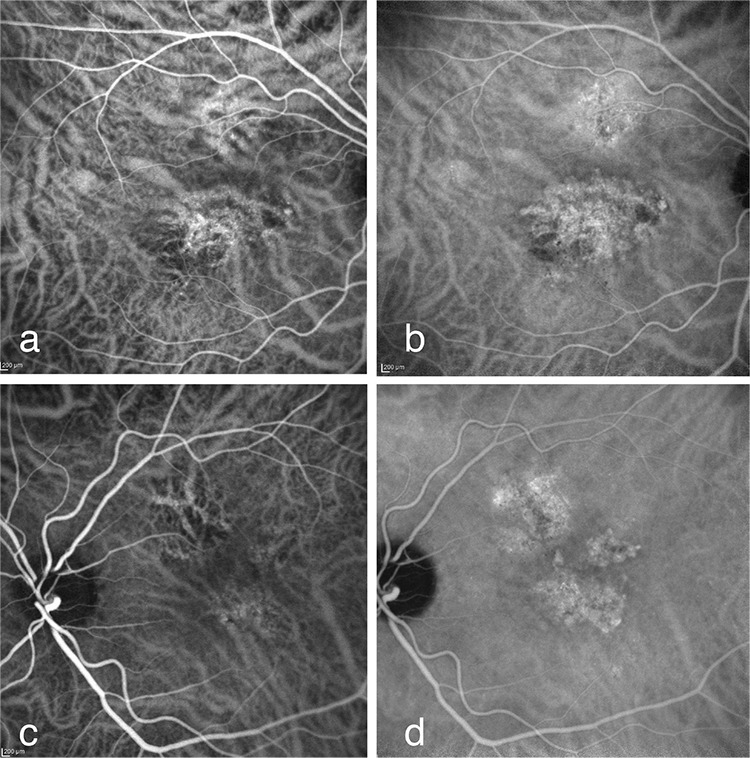
a) The appearance of dilated choroidal vessels and hyperfluorescence in the central area starting at second 28 of indocyanine green angiography in the right eye; b) plaque choroid neovascularization with contours clarifying in the central area and having polypoid expansion in the nasal at 4 minutes, 47 seconds of indocyanine green angiography in the right eye; c) dilated choroidal vessels in the macula at 37 seconds of indocyanine green angiography in the left eye; d) two plaques of choroid neovascularization in the subtemporal and temporal parafoveal areas and retinal pigment epithelium irregularity and hyperflorescence due to atrophy in the superior parafoveal area at 8 minutes, 39 seconds of indocyanine green angiography in the left eye

On ICGA, both eyes were found to have dilated choroidal vessels in the early phase and appearance consistent with plaque CNV in the late phase (Figure 2).

**Figure 3:**
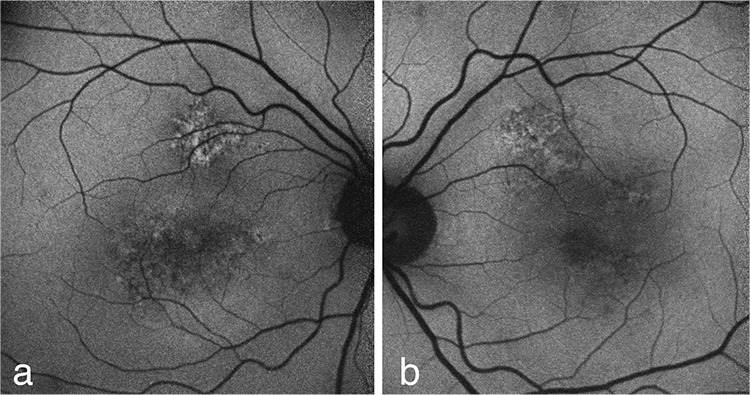
Hypo and hyper signal changes in fundus autofluorescence; a) in foveal and superior parafoveal areas of the right eye; b) and in the superior and temporal parafoveal areas of the left eye

Fundus autofluorescence revealed hyperautofluorescent spots were seen in the central fovea and superonasal to the fovea (Figure 3).

**Figure 4:**
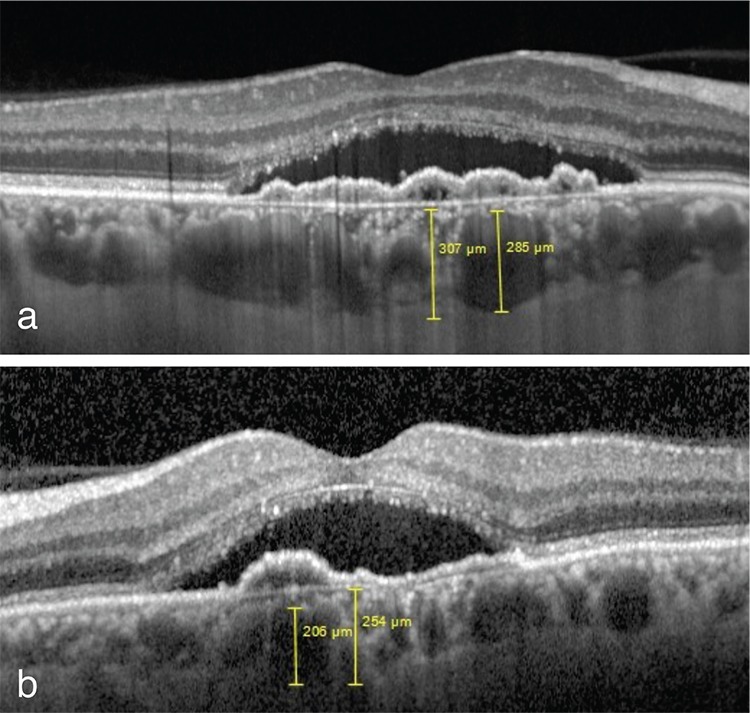
Shallow irregular pigment epithelium detachment, subretinal fluid and pachy-veins on spectral domain optical coherence tomography; a) subfoveal choroid thickness and pachy-vein thickness are 307 μm and 285 μm in the right eye; b) 254 μm and 206 μm in the left eye, respectively

Bilateral subretinal fluid, shallow irregular PED, and pachy-veins were observed on spectral domain OCT (Heidelberg). Subfoveal choroid thickness was 307 µm in the right eye and 254 µm in the left. Pachy-vein thickness was measured as 285 µm in the right eye and 206 µm in the left (Figure 4).

**Figure 5:**
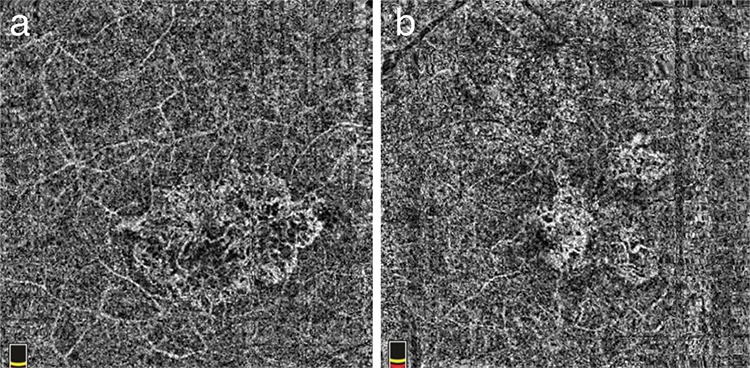
a) Optical coherence tomography angiography image of a single vascular network of type 1 choroid neovascularization; b) optical coherence tomography angiography image of 2 vascular network of type 1 choroid neovascularization

OCT-A (RTVue XR “Avanti”, Optovue, Fremont, California, United States of America) imaging revealed tangled hyperreflective neovascular network compatible with type 1 CNV in the choroid slab of both eyes. The selected CNV area was 4.671 mm^2^ in the right eye and 3.533 mm^2^ in the left. The flow area through the selected CNV area was 2.847 mm^2^ in the right eye and 2.211 mm^2^ in the left. The largest diameter of the selected CNV area was 1.26 mm in the right eye and 1.28 mm in the left (Figure 5).

## Discussion

PNV was first described by Pang and Freund^8^ in 2015. The disease may consist of type 1 CNV that develops secondary to central serous chorioretinopathy or pachychoroid pigment epitheliopathy. PNV should be suspected in cases of thickened choroid with type 1 CNV without characteristic findings of age-related macular degeneration (AMD) such as drusen or hemorrhages. It is characterized by the presence of shallow, irregular PED.

In a study by Miyake et al.^9^ including 200 patients diagnosed with PNV and AMD, 19.5% of the cases were diagnosed with PNV. Subfoveal choroid thickness was found to be greater in patients with PNV than in those with AMD. They reported that genetic mutations were detected less frequently in patients with PNV. In addition, PNV was observed in younger patients compared to AMD.^10^

The etiopathogenesis of pachychoroid spectrum diseases involves microtrauma to the Bruch’s membrane from the enlarged pachy-veins in the Haller’s layer. This causes choriocapillaris loss and RPE changes. Neovasculopathy develops as a result of extension of neovascularization beneath the RPE.^1^

FFA and ICGA are used in the diagnosis of CNV and are known to cause nausea and anaphylaxis in rare cases.^11^ OCT-A enables image acquisition by serial OCT scanning and is a reliable method that allows imaging of retinal and choroidal vasculature without needing any dye injection.^7^ The tangled vascular network under the shallow irregular PED can be imaged with OCT-A. In a study including 16 patients (22 eyes) with shallow irregular PED, CNV was detected in 95% of the patients with OCT-A. Compared to other angiography techniques, OCT-A is shown to be more successful in demonstrating type 1 CNV.^12^

Similarly, in the present case we observed shallow PED, subretinal fluid, thickened choroid, and pachy-veins on spectral domain OCT and appearance consistent with type 1 CNV in FFA and ICGA. OCTA showed a CNV network in the areas corresponding to the type 1 CNV observed on ICGA. In a case series by Azar et al.^13^ including 5 PNV patients, the presence of neovascularization could not be fully identified with FFA and ICGA in 2 patients, whereas the presence of tangled filamentous vascular network was detected in all of the patients with OCT-A. Therefore, these findings indicate that OCT-A can detect CNV before FFA and ICGA in pachychoroid spectrum diseases.

In conclusion, as we have also observed in our case, non-invasive OCT-A imaging generally supports fundus angiography images with regard to the diagnosis of type 1 CNV in PNV. OCT-A should be used in combination with other methods for the detection of vascularization in AMD presenting with shallow PED and in pachychoroid spectrum diseases.
